# Cardio-Cerebral Protective Effect of Moxibustion on Phlegm-Dampness Type Hypertension: Protocol for a Randomized Controlled Trial

**DOI:** 10.2196/79158

**Published:** 2025-12-29

**Authors:** Ling Cheng, Ning Bai, Liang Zheng, Lulu Cao, Huangan Wu, Ruiping Wang, Chen Zhao, Yunli Shen, Haiyin Zhao, Gang Li, Bing Yang, Qinghui Yang, Yan Xing, Jianhong Cao, Yixing Wang, Ling Xu, Junjie Yan, Jie Cao, Yiyang Guo

**Affiliations:** 1 Acupuncture and Moxibustion Department of East Hospital Affiliated to Tongji University Shanghai China; 2 Shanghai Jinshan Traditional Chinese Medicine-Integrated Hospital Shanghai China; 3 State Key Laboratory of Cardiovascular Diseases and Medical Innovation Center, Shanghai East Hospital, School of Medicine, Tongji University Shanghai China; 4 Shanghai Research Institute of Acupuncture and Meridian Shanghai China; 5 Shanghai Skin Disease Hospital, Medical School, Tongji University Shanghai China; 6 Shanghai University of Traditional Chinese Medicine Shanghai China; 7 Longhua Hospital Shanghai University of Traditional Chinese Medicine City China

**Keywords:** hypertension, blood pressure, moxibustion, acupoint specificity, cardioprotection, moxibustion, treatment, patients, trials

## Abstract

**Background:**

Hypertension is associated with a high rate of disability and mortality and leads to a substantial socioeconomic burden. Moxibustion is an external treatment in traditional Chinese medicine that has been used to treat mild to moderate hypertension in individuals with phlegm-dampness constitution and has demonstrated acupoint specificity. However, a standard large-scale randomized controlled trial is still needed to verify its effectiveness. This study is proposed to examine the clinical effectiveness and potential cardioprotective benefits of moxibustion performed at home as a treatment for individuals with phlegm-dampness hypertension.

**Objective:**

The objective of this trial is to evaluate the cardio-cerebral protective clinical efficacy of moxibustion for phlegm-dampness type hypertension and to explore its acupoint specific effects.

**Methods:**

This study is a multicenter, randomized controlled trial. A total of 120 patients with mild to moderate hypertension and phlegm-dampness constitution will be recruited and randomly assigned in a 1:1 ratio to the treatment group (acupoint: Zusanli, ST36) or the control group (acupoint: Xuanzhong, GB39). All patients will receive 12 weeks of treatment and a 12-week follow-up period. The primary outcome measure is the change in morning systolic blood pressure from baseline to week 12. The secondary outcome measures include blood pressure–related indicators (morning diastolic blood pressure, average systolic blood pressure, average diastolic blood pressure, nighttime systolic blood pressure, nighttime diastolic blood pressure, and blood pressure circadian rhythm) and short-term blood pressure variability coefficient, all of which will be measured by 24-hour ambulatory blood pressure monitoring. Additionally, cardiac-related indicators measured by 24-hour Holter monitoring, metabolic disorder-related indicators, liver and kidney function indicators, transformed scores of the traditional Chinese medicine phlegm-dampness constitution scale, and the Montreal Cognitive Assessment will also be evaluated.

**Results:**

This study was registered on July 5, 2024, with the Chinese Clinical Trial Registry. Data collection began in June 2023 and ended in February 2025. Currently, data from this trial are in the collection phase, and no data analysis has been performed. As of January 2025, we have collected data from 118 patients. The results of this trial are expected to be submitted for publication in May 2026.

**Conclusions:**

This multicenter, randomized, controlled clinical trial will provide evidence on the clinical effectiveness and potential cardioprotective benefits of moxibustion performed at home as a treatment for individuals with phlegm-dampness type of hypertension.

**Trial Registration:**

Chinese Clinical Trial Registry ChiCTR2400086582; https://www.chictr.org.cn/showproj.html?proj=211688

**International Registered Report Identifier (IRRID):**

DERR1-10.2196/79158

## Introduction

### Background

Hypertension is a chronic disease characterized by persistently elevated arterial blood pressure in the systemic circulation. It is a major risk factor for premature death, stroke, ischemic heart disease, and other cardiovascular and renal diseases globally [1]. Hypertension imposes a substantial global health burden [2], affecting approximately one-third of adults, with only up to 54% receiving a diagnosis [3]. China is among the countries with the highest increase in hypertension prevalence and the lowest control rates. In 2018, the prevalence of hypertension among Chinese adults was 27.5%, whereas the control rate was only 15.3% [4]. Poor long-term blood pressure control can cause irreversible damage to multiple target organs, including the heart, brain, kidneys, and blood vessels, leading to increased disability and mortality rates. Therefore, early intervention is crucial to slow the progression of hypertension and reduce the associated social and economic burdens.

Currently, the primary approaches for hypertension treatment include lifestyle modification and medications. The medication adherence of hypertensive patients in China is only 42.5% [4], far lower than the average level in Asian countries, which is a significant factor contributing to low blood pressure control rates [5], increasing the risk of cardiovascular and target organ damage [6], and negatively impacting patients’ quality of life [7]. Thus, there is an urgent need to develop a treatment method that can maintain stable blood pressure with high safety, good adherence, tolerability, and cost-effectiveness.

Moxibustion, a traditional Chinese medicine (TCM) therapy, uses thermal effects, infrared radiation, photobiochemical effects, and bioelectric effects produced by burning mugwort to stimulate specific acupoints and meridians, thereby dredging meridian, promoting blood circulation, and regulating the body’s neuroendocrine-immune functions [8]. Compared with medication alone, the additional application of moxibustion can effectively control blood pressure [9], correct imbalanced body constitutions [10], and significantly alleviate related symptoms, such as dizziness, headache, and insomnia [11]. Moxibustion is characterized by rapid onset, stable efficacy [12], improved cognitive function [13], and fewer side effects with high safety [11]. Its mechanism of action is multifaceted and multitargeted [13].

Our previous studies indicate the acupoint specificity of moxibustion’s antihypertensive effects [14-16], with differential activation of arcuate neurons depending on the targeted acupoint. Specifically, nerve input at Zusanli (ST36) is greater than that at Xuanzhong (GB39) [17-19]. Clinical trials have shown that moxibustion at Zusanli can significantly reduce 24-hour average systolic blood pressure and improve myocardial oxygen consumption, whereas moxibustion at Xuanzhong shows no significant effect [15]. Although we have confirmed the efficacy and acupoint specificity of moxibustion in treating hypertension, a standard large-sample clinical trial is still needed.

### Objective

We designed this multicenter randomized controlled trial (selecting Zusanli [ST36] as the treatment group and Xuanzhong [GB39] as control group) to provide clinical evidence for the acupoint specific effects of moxibustion on hypertension and its protective effects on the heart and brain.

## Methods

### Ethical Considerations

This study protocol was approved by the Ethics Committee of the Shanghai Tongji University-affiliated Oriental Hospital (2023 research review: 145), the Shanghai University of Traditional Chinese Medicine-affiliated Longhua Hospital (2024LCSY161), and the Shanghai Acupuncture and Meridian Research Institute (2023-169). Written informed consent was obtained from all participants prior to the study and data publication. All study-related examinations and treatments will be provided to participants free of charge. No additional compensation will be offered. The study was registered on July 5, 2024, with the Chinese Clinical Trial Registry (ChiCTR2400086582). All participants will be provided with detailed study information and must provide written informed consent prior to enrollment. The trial results will be submitted for publication in a peer-reviewed journal. Neither patients nor the public were involved in the study's design, conduct, reporting, or dissemination plans.

### Case Selection

Participants in this trial are outpatients receiving treatment between June 2023 and February 2025 at the Oriental Hospital affiliated with Tongji University, Longhua Hospital affiliated with Shanghai University of Traditional Chinese Medicine, and the Shanghai Research Institute of Acupuncture and Meridian. All patients are recruited and screened based on the inclusion and exclusion criteria outlined in the study.

### Inclusion Criteria

In this study, the inclusion criteria are as follows: (1) patients diagnosed with grade 1 or 2 hypertension according to the “Chinese Guidelines for the Prevention and Treatment of Hypertension” (2018 revision) [20] (Multimedia Appendix 1); (2) patients with phlegm-dampness constitution according to the “Classification and Determination of TCM Constitution” (ZYYXH/*t* 157-2009) [21] (Multimedia Appendix 2); (3) age between 18 and 75 years, regardless of sex (male or female); (4) no contraindications to moxibustion: (5) without secondary hypertension, hyperthyroidism, or without severe heart conditions (eg, atrial fibrillation and moderate valvular heart disease), and not being in the unstable phase of angina pectoris or myocardial infarction, not being in the acute phase of stroke, and no malignant diseases; and (6) willingness to participate in the study and to sign the informed consent form.

### Exclusion Criteria

In this study, the exclusion criteria include the following: (1) patients with severe mental disorders (eg, depression, anxiety, or schizophrenia), or with severe conditions requiring hospitalization or interventions; (2) pregnant or breastfeeding women; (3) patients with severe heart, brain, liver, or kidney diseases; and (4) patients unable to tolerate and to persist with moxibustion treatment.

### Elimination Criteria

Participants who do not adhere to the study requirements during the trial, and those who do not meet the inclusion criteria after enrollment, will be removed from this study.

### Dropout Criteria

In this study, the dropout criteria include the following: (1) participants who meet the inclusion criteria but do not complete the trial or who withdraw midway for various reasons; (2) participants who do not follow the prescribed treatment plan or who receive other treatments or medications that interfere with the assessment of efficacy; and (3) participants who are lost to follow-up or die before completing the trial.

### Termination Criteria

In this study, patients who experience severe adverse reactions or develop severe issues during the trial (eg, sudden exacerbation of preexisting conditions or new severe diseases) that make it impossible to assess efficacy will be advised to discontinue the trial.

### Recruitment

In this study, we disseminate study information through official social media, flyers, and online articles in hospitals and communities for patients’ reference. Interested patients can register online and based on proximity, visit designated clinical sites for screening. After confirmation by clinical physicians and after signing the informed consent form, eligible participants will be randomly assigned to a treatment group or a control group in a 1:1 ratio and will receive 12 weeks of moxibustion treatment, followed by 12 weeks of follow-up. All procedures will strictly adhere to the Declaration of Helsinki.

### Sample Size Calculation

The primary efficacy evaluation index for this study is the morning systolic blood pressure measured by 24-hour ambulatory blood pressure monitoring (ABPM). The sample size was estimated based on statistical methods. Preliminary trial data showed that the treatment group had a 7.2 (SD 3.3) mm Hg greater reduction in 24-hour morning systolic blood pressure than the control group. In this study, we apply the superiority design and set the superiority margin (Δ) as 5 mm Hg [22], with a significance level of α equal .025 (1-sided) and a power of β equal .1. The mean value for treatment improvement between the treatment and control groups is set at 7.2 (SD 3.3) mm Hg, with a SD (σ) of 3.3 mmHg, based on the formula for comparing means in a superiority trial [22]:



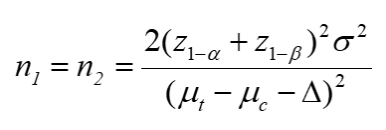



The primary efficacy endpoint of this study was the change in morning systolic blood pressure as measured by 24-hour ABPM. Sample size determination was based on a superiority design for comparing two independent means. The calculation incorporated the following parameters: an expected between-group difference (μ₁–μ₂) of 7.2 mm Hg derived from preliminary data, a common SD (σ) of 3.3 mm Hg, a prespecified superiority margin (Δ) of 5 mm Hg [22], a one-sided α level of 0.025, and 90% statistical power (1–β). These values were applied to the standard sample size formula for superiority trials to ensure adequate power for detecting a clinically meaningful treatment effect.

### Randomization Method

Allocation concealment was ensured with sequentially numbered, opaque, sealed envelopes. An independent statistician generated the allocation sequence using SAS software (version 9.4; SAS Institute), which assigned participants in a 1:1 ratio to the treatment group (acupoint ST36) or the control group (acupoint GB39). The assignments were sealed in tamper-evident envelopes and stored securely at the coordinating center. Following participant enrollment, the study coordinator retrieved and opened the next sequential envelope to reveal the assignment to the intervention clinician, thereby ensuring complete concealment and unpredictability of the allocation for researchers and participants.

### Blinding

Multiple safeguards were implemented to maintain blinding in this self-administered moxibustion trial. Participants were blinded by being told they “might receive different acupoint treatments” during consent and were assigned only a unique ID. Cross-contact was prevented through segregated scheduling. To ensure consistency and control performance bias, a single clinician (uninvolved in the assessment) provided standardized training to all participants. Data collectors, outcome assessors, and statisticians were blinded, with unblinding permitted only for serious adverse events, which would lead to separate analysis of that participant’s data.

### Intervention Protocol

#### Basic Treatment

After enrollment, participants will continue their previous medication and record it in the case report form.

#### Treatment Group

Participants in the treatment group will receive moxibustion at Zusanli (ST36). All participants will undergo training by professionals on acupoint location, moxibustion techniques, and precautions before beginning treatment. Only patients who pass the training and evaluation will be allowed to perform moxibustion at home, 3 times a week for 12 weeks. The first moxibustion session will be conducted under professional supervision in the clinic, with the skin temperature at the moxibustion site recorded before and after the session. Subsequently, participants will document each moxibustion session with photographs and will complete a moxibustion record form, noting the date, duration, and any adverse reactions. Researchers will conduct 2 random follow-up visits per week to remind participants to complete their moxibustion sessions on time and to verify the frequency, location, duration, and completion of the sessions using photos and record forms. Participants will also undergo outpatient evaluations every 2 weeks to assess their moxibustion adherence and will receive comprehensive assessments at baseline, week 4, week 12, and week 24.

#### Control Group

**Figure 1:**
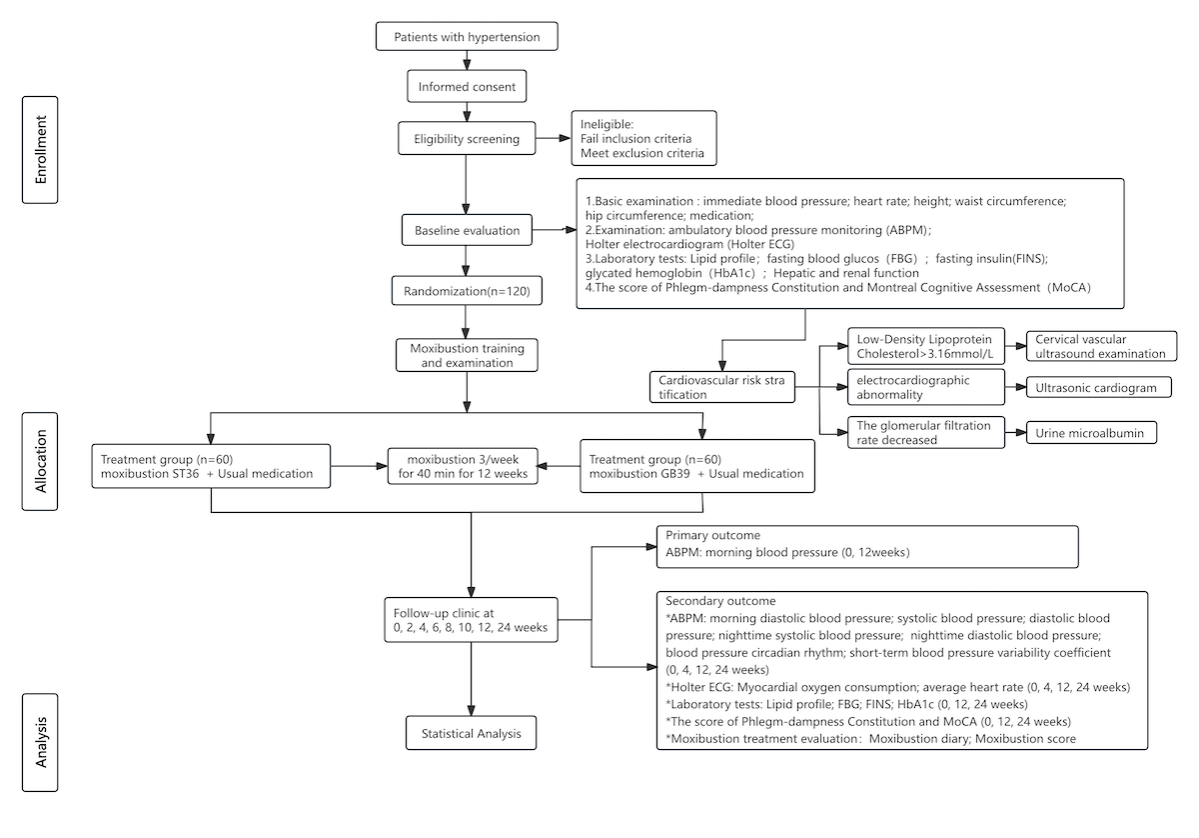
Flow diagram of the trial procedure. ABPM: ambulatory blood pressure monitoring; FBG: fasting blood glucose; FINS: fasting insulin; HbA1c: glycated hemoglobin; HDL: high-density lipoprotein; LDL: low-density lipoprotein; TC: total cholesterol; MoCA: Montreal Cognitive Assessment.

Participants in the control group will receive moxibustion at Xuanzhong (GB39). The training, evaluation, and assessment procedures will be identical to those of the treatment group to complete the study according to the design process (Figure 1). The time points of the outcome measures are summarized in Table 1.

**Figure 2:**
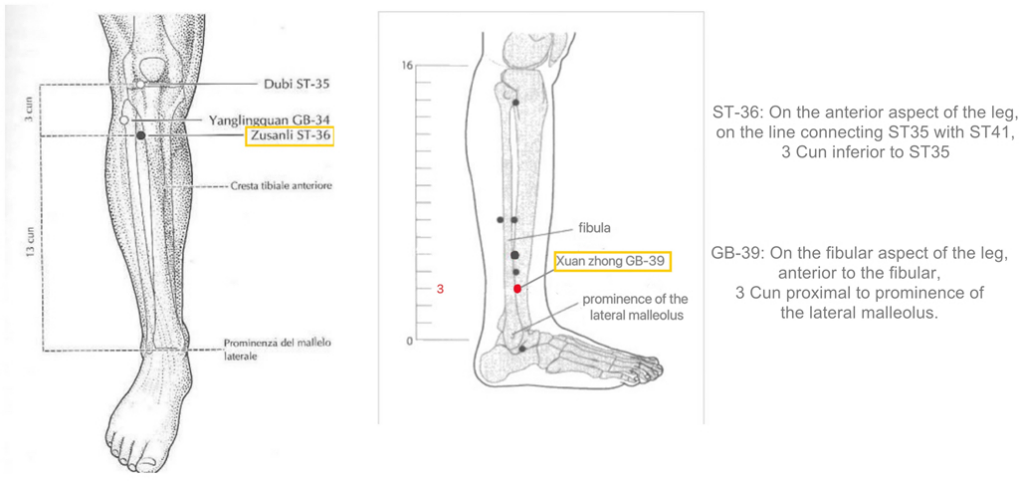
Acupuncture point diagram for the location of the acupoints.

Acupoint locations in this study are determined according to the “Names and Locations of Acupoints” (GB/T12346-2021) standard (Figure 2).

**Table 1:** Time points of the outcome measures.

Assessment	Enrollment and baseline	Allocation		After allocation						Closeout
		0 wk	2 wk	4 wk	6 wk	8 wk	10 wk	12 wk	24 wk	
Informed consent	✓	—^a^	—	—	—	—	—	—	—	—
Inclusion or exclusion	✓	—	—	—	—	—	—	—	—	—
General information	✓	—	—	—	—	—	—	—	—	—
Random allocation		✓	—	—	—	—	—	—	—	—
Intervention	—	✓	✓	✓	✓	✓	✓	✓		—
Concomitant medication	✓	—	✓	✓	✓	✓	✓	✓	✓	—
24 h ABPM^b^	✓	—	—	✓	—	—	—	✓	✓	—
24 h Holter ECG^c^	✓	—	—	✓	—	—	—	✓	✓	—
Lipid profile (Triglycerides, TC^d^, HDL^e^, and LDL^f^)	✓	—	—	✓	—	—	—	✓	✓	—
Glycemic markers (FPG^g^, FINS^h^, HbA_1c_^i^)	✓	—	—	—	—	—	—	✓	✓	—
^Hepatic and renal function^	✓	—	—	—	—	—	—	✓	✓	—
^Phlegm-damp constitution^	✓	—	—	—	—	—	—	✓	✓	—
Cardiovascular risk stratification	✓	—	—	—	—	—	—	✓	✓	—
MoCA^j^	✓	—	—	—	—	—	—	✓	✓	—
Adverse events and other unintended effects	—	—	✓	✓	✓	✓	✓	✓	✓	—
^Compliance monitoring^	—	—	✓	✓	✓	✓	✓	✓	✓	—
^Completion summary^	—	—	✓	✓	✓	✓	✓	✓	✓	—

^a^Not available.

^b^ABPM: ambulatory blood pressure monitoring.

^c^Holter ECG: Holter electrocardiogram.

^d^TC: total cholesterol.

^e^HDL: high-density lipoprotein.

^f^LDL: low-density lipoprotein.

^g^FBG: fasting blood glucose.

^h^FINS: fasting insulin.

^i^HbA_1c_: glycated hemoglobin.

^j^MoCA: Montreal Cognitive Assessment.

#### Materials and Tools

The material and tools used for moxibustion in this study will include (1) Lei Huo moxibustion device (Registration Number: 20,232,200,237; Hunan Cihui Medical Technology Co, Ltd); (2) OMRON YE-680 series digital blood pressure monitor (Registration Number: 20,202,071,574; Omron Healthcare Co, Ltd); (3) RGT-120/160 weighing scale (Changzhou Wujin Weighing Apparatus Co, Ltd); and (4) 150 cm measuring tape.

### Assessment and Outcomes

#### Overview

Participants will undergo evaluations at weeks 0, 2, 4, 6, 8, 10, 12, and 24 during outpatient visits. Twenty-four-hour ambulatory blood pressure and 24-hour Holter electrocardiogram will be examined at weeks 0, 4, 12, and 24. General information (weight, height, waist circumference, hip circumference, family history, medical history, concomitant medications, and lifestyle behavior), relevant laboratory tests (4 items of lipids, fasting blood glucose, glycosylated hemoglobin, serum insulin, homeostatic model assessment of insulin resistance, homeostatic model assessment of β-cell function, insulin-sensitivity index, triglyceride-glucose index, metabolic score for insulin resistance, liver function, and kidney function), heart function, vascular risk stratification, and scale assessment (TCM phlegm-dampness physique score and the Montreal Cognitive Assessment) will be examined at weeks 0, 12, and 24. Major unconscionable cerebrovascular injury, target organ injury, and moxibustion adverse events will be recorded during the study period.

#### Primary Outcome Measure

In this study, the primary outcome measure is the morning systolic blood pressure, which will be measured by 24-hour ABPM (assessment at weeks 0 and 12).

#### Secondary Outcome Measures

##### Blood Pressure–Related Indicators (Measured by 24-Hour ABPM)

Morning diastolic blood pressure, average systolic blood pressure, average diastolic blood pressure, nighttime systolic blood pressure, nighttime diastolic blood pressure, and circadian rhythm of blood pressure will be assessed (at weeks 0, 4, 12, and 24). Twenty-four-hour diastolic blood pressure, 24-hour nocturnal systolic blood pressure, 24-hour nocturnal diastolic blood pressure, 24-hour morning systolic blood pressure, 24-hour morning diastolic blood pressure, circadian rhythm of blood pressure, 24-hour systolic blood pressure variability, and 24-hour diastolic blood pressure variability will also be assessed at different time points (at weeks 0, 4, 12, and 24)

##### Cardiac-Related Indicators (Measured by 24-Hour Holter Electrocardiogram)

Total heartbeats, myocardial oxygen consumption, average heart rate, heart rate variability (HRV) parameters (ie, percentage of successive NN intervals that differ by more than 50 ms, HRV triangular index, root mean square of successive differences, SD of normal-to-normal [SDNN] intervals, SD of the average normal-to-normal intervals, SDNN index, low-frequency [LF] power, high-frequency [HF] power, and the LF and HF ratio) will be assessed at weeks 0, 4, 12, and 24.

##### Laboratory Tests

Triglycerides, cholesterol, high-density lipoprotein, low-density lipoprotein, fasting blood glucose, glycated hemoglobin, fasting insulin, homeostatic model assessment of insulin resistance, homeostatic model assessment of β-cell function, insulin-sensitivity index, triglyceride-glucose index, metabolic score for insulin resistance, and liver and kidney function will be measured at weeks 0, 12, and 24.

##### Scale Evaluations

The TCM phlegm-dampness constitution score, Montreal Cognitive Assessment will be administered at weeks 0, 12, and 24.

#### Exploratory Outcomes

After enrollment, participants will be stratified based on cardiovascular risk using a general information survey designed according to the “Chinese Guidelines for the Prevention and Treatment of Hypertension” (2018 revision [20]) as in Multimedia Appendix 3.

For participants with abnormal Holter electrocardiogram results (eg, Sokolow-Lyon voltage exceeding 3.8 mV or Cornell product exceeding 244 mV·ms), a comprehensive echocardiogram will be performed.

For participants with elevated low-density lipoprotein levels (ie, above 3.16 mmol/L), carotid ultrasound will be conducted to compare vascular characteristics and plaque changes before and after treatment.

For participants with decreased glomerular filtration rate (ie, <59 mL/min/1.73 m^2^) or mildly elevated serum creatinine (male: >115 μmol/L [>1.3 mg/100 mL]; female: >107 μmol/L [>1.2 mg/100 mL]), microalbuminuria will be assessed.

#### Moxibustion Adherence and Completion

A comprehensive strategy will be used to optimize adherence to home-based moxibustion. Before the intervention, participants will receive standardized training via an instructional video and will be instructed to log details (duration, temperature, and adverse events) as in Multimedia Appendix 4 and media (video or photo) for each session. During the study period, adherence will be promoted through irregular WeChat messages and telephone follow-ups for reminders, supplemented by unannounced random video checks to verify the participants’ procedures. Furthermore, biweekly follow-up visits will be conducted at the research center. These sessions will comprise a face-to-face review of moxibustion logs and video or photo documentation, an assessment of procedural technique and acupoint localization accuracy (Multimedia Appendix 5), and the exchange of used moxa sticks for new supplies.

#### Safety Evaluation

Major adverse cardiovascular and cerebrovascular events, liver function (alanine aminotransferase and aspartate aminotransferase) and kidney function (blood urea nitrogen and creatinine) and moxibustion-related adverse events (burns, blisters, dizziness during moxibustion, ulceration, infection, and local abscesses at the moxibustion site; other discomforts after moxibustion may include dizziness, fatigue, allergies, and loss of appetite) will be recorded in this study. Moreover, adverse events will be monitored through patient self-reports and assessments by health care professionals during the study. Participants will be instructed to immediately report any adverse events to their supervising clinician, who will determine whether further examinations or treatments are necessary and will provide timely interventions. For serious adverse events, the trial will be immediately halted. All adverse reactions, their severity, duration, and any actions taken will be thoroughly documented.

### Data Management and Quality Control

All data related to study participants will be stored and backed up according to trial standards. Case report forms will be uniformly printed after they are designed by the research team, and all trial data for participants will be accurately recorded on these forms. Paper-based data will be retained for at least 3 years. Additionally, an independent data safety monitoring committee (DSMC) will be established before the trial, comprising 3 experts with backgrounds in cardiology, acupuncture, and statistics. The DSMC will hold online meetings every 4 months to oversee trial progress and review data safety and quality. A midterm analysis will be conducted at the halfway point of participant enrollment, and the DSMC will determine whether the trial needs to be terminated early. Personal privacy information (eg, name, age, and phone number) will be anonymized in DSMC reports.

### Statistical Analysis

Data analysis of the primary outcome will be based on the full analysis set constructed by the intention-to-treat principle, whereby patients will remain in their original group regardless of contamination. The per-protocol set will also be analyzed using sensitivity analysis for the primary outcome to evaluate whether contamination affects the direction of the effect. The secondary outcomes will also be compared based on the per-protocol set. The need to impute missing values will be determined by the proportion of and reasons for missing data. If imputation is necessary, appropriate methods, such as the last observation carried forward, the “best-worst-case” scenario, and multiple imputation regression modeling, will be chosen according to the distribution of the missing values.

Potential center effects will be statistically addressed through mixed-effects modeling, notwithstanding standardized central screening and intervention procedures. For primary continuous outcomes, linear mixed-effects models will incorporate the treatment group as a fixed effect and center as a random effect, with center-specific random intercepts accounting for recruitment-related heterogeneity. Secondary outcomes will be analyzed using analogous generalized linear mixed models. The primary analysis will use an intention-to-treat approach. The changes in the primary outcome and other secondary outcomes will be compared by using a linear mixed-effects model with group-by-time point interaction (baseline to week 12). For outcomes measured at multiple time points, a repeated-measures ANOVA will first test for overall group, time, and interaction effects. Upon finding a significant interaction (exact *P* values will be reported), post hoc comparisons will be conducted using a hierarchical adjustment strategy: Benjamini-Hochberg false discovery rate control for the primary outcome across time points and Bonferroni correction for families of secondary outcomes. Sociodemographic and clinical characteristics at baseline will be examined for potential group differences by 2-tailed *t* test or χ^2^ test, with exact *P* values reported. Compliance will be presented using descriptive statistics. The specified significance levels (0.025 for the primary outcome and 0.05 for all other analyses) represent the α thresholds (*P* value cutoffs) for hypothesis testing. All *P* values will be reported per journal style: expressed to two digits (or three if *P*<.01) and as *P*<.001 if below that threshold. Data analysis was conducted with SPSS (version 28.0; IBM Corp).

## Results

The study was registered on July 5, 2024, with the Chinese Clinical Trial Registry (ChiCTR2400086582). Data collection began in June 2023 and ended in February 2025. Currently, data from this trial are in the collection phase, and no data analysis has been performed. As of January 2025, we have collected data from 118 patients. The results of this trial are expected to be submitted for publication in May 2026.

## Discussion

### Anticipated Findings

This study aims to explore the acupoint specificity and clinical efficacy of moxibustion in treating hypertension and its protective effects on the heart and brain. In this study, we employed the Montreal Cognitive Assessment to quantitatively evaluate cognitive protection. Hypertension contributes to cerebral microvascular injury and silent cerebral small vessel disease, which predominantly impairs cognitive domains sensitively captured by the Montreal Cognitive Assessment, including executive function and attention. By streamlining acupoint selection and establishing a standardized home-based moxibustion protocol, this study seeks to offer patients with hypertension a safe, convenient, and adherent treatment option. This approach may help patients develop healthy lifestyle habits, contributing positively to the improvement of their quality of life and the prevention of cardiovascular and cerebrovascular diseases.

In TCM, body constitution is considered a determining factor in disease onset and progression. In today’s fast-paced society, factors such as high stress, poor dietary habits, and lack of exercise contribute to the development of a phlegm-dampness constitution. Research has shown that this constitution is a high-risk factor for hypertension and its related complications. Patients with phlegm-dampness hypertension are more prone to insulin resistance, which increases the risk of target organ damage [23], leads to a higher cardiovascular risk over 10 years, and is associated with a poorer prognosis [24]. Molecular genetics research indicates that genes expressed in individuals with phlegm-dampness constitution are similar to those associated with obesity and metabolic syndrome [25]. Therefore, improving phlegm-dampness constitution is crucial for controlling hypertension and preventing cardiovascular and cerebrovascular diseases [26].

The Zusanli acupoint (ST36) is a He-Sea point and lower He-Sea point of the Stomach Meridian, known for its functions of regulating Qi and blood, harmonizing the stomach, and eliminating dampness. X-ray phase-contrast imaging studies have shown that the 3D structure of Zusanli has a higher density of local blood vessels [27], mast cells [28], and prokineticin receptor 2–expressing cells [29] compared with nonacupoints or other acupoints, along with accumulation of elements like calcium, iron, and zinc [30]. The capsaicin receptor (TRPV1) is abundantly present in the mast cells at Zusanli [31], and moxibustion heat at 43 to 46 °C can activate TRPV1 ion channels, mediating mast cell degranulation and releasing bioactive substances that exert antihypertensive effects [32]. Additionally, moxibustion at Zusanli can inhibit immune-inflammatory responses, counter oxidative stress, regulate mitochondrial function and cellular autophagy, enhance blood circulation, and regulate metabolic disorders. Our preliminary clinical observations indicated that although moxibustion at Xuanzhong (GB39) did not significantly lower blood pressure, it did improve metabolic disorders and sleep quality. Therefore, this trial uses Xuanzhong as the control group (rather than a blank or nonacupoint control) to observe the differential efficacy of the 2 acupoints while helping participants develop good moxibustion habits, providing an effective way to prevent and manage chronic diseases [13].

Acute cardiovascular and cerebrovascular events frequently occur in the morning, and numerous clinical studies have confirmed that morning hypertension is an independent predictor of such events [2], positively correlating with their incidence and mortality [33]. For every 1 mm Hg increase in morning blood pressure, the risk of cardiovascular-related death increases by 2.1% [34,35]. Compared with patients with morning blood pressure below 125 mm Hg, those with morning hypertension have significantly higher risks of stroke [36] (hazard ratio 6.01) and coronary events (hazard ratio 6.24). Stroke risk is positively correlated with morning systolic blood pressure levels, with hazard ratios of 2.45, 2.80, 3.58, and 6.52 for morning systolic blood pressure ranges of 135 to 144 mm Hg, 145 to 154 mm Hg, 155 to 164 mm Hg, and ≥165 mm Hg, respectively [37]. Regardless of clinical blood pressure levels, patients with morning home systolic blood pressure ≥145 mm Hg have higher cardiovascular risk (hazard ratio 2.47) than those with levels <125 mm Hg [38]. Morning hypertension is also a significant cause of vascular cognitive impairment [39]. It is an independent risk factor for the 10-year incidence of atherosclerotic cardiovascular disease [40]. Patients with nondipper (0%-10%) or reverse-dipper (<0%) nocturnal blood pressure rhythms have higher risks of cardiovascular events and all-cause mortality [41], as well as a greater likelihood of reduced glomerular filtration rate and elevated creatinine levels [42]. Blood pressure variability, which reflects fluctuations in blood pressure within a certain range, is an important indicator of blood pressure stability. Individuals with higher short-term blood pressure variability, as measured by 24-hour ABPM, are more likely to develop subclinical organ damage and adverse cardiovascular events [43].

Autonomic nervous dysfunction can lead to altered circadian rhythms of blood pressure and increased blood pressure variability, exacerbating damage to vascular endothelium and arterial baroreceptors, accelerating vascular wall fibrosis and atherosclerosis, and ultimately causing target organ damage [44]. HRV, which measures differences between adjacent heartbeats, reflects autonomic nervous activity and inflammation, and is related to coronary physiology [45] and the severity of coronary artery disease [36]. Reduced HRV is closely associated with various cardiovascular diseases, diabetic microvascular complications, and imbalanced body constitutions [46]. SDNN, SD of the average normal-to-normal intervals, root mean square of successive differences, LF, and HF values were significantly lower in patients with hypertension and poorly controlled blood pressure and were independent risk factors for uncontrolled blood pressure [36]. Additionally, prolonged elevated arterial blood pressure increases myocardial oxygen consumption and cardiac workload, leading to myocardial ischemia and cardiovascular diseases, such as coronary heart disease and left ventricular hypertrophy. Evaluating total heartbeats, average heart rate, myocardial oxygen consumption, HRV indices, ischemic ST-T changes, and arrhythmias through 24-hour Holter electrocardiogram is crucial for the early diagnosis of occult coronary artery disease and the prevention and management of acute cardiovascular diseases [36].

Insulin resistance is a common pathophysiological mechanism underlying hypertension, type 2 diabetes, metabolic syndrome, and cardiovascular diseases [47]. It often leads to metabolic, structural, and functional abnormalities in organs and tissues, such as microvascular disease and atherosclerosis, and is an independent risk factor for cardiovascular diseases [48]. The metabolic score for insulin resistance index, which integrates fasting blood glucose, lipids, and BMI, is a novel insulin index that significantly correlates with the incidence of hypertension, angina, heart failure, and myocardial infarction, making it a risk factor for coronary artery disease and the severity of coronary artery lesions [49].

### Limitations and Future Directions

The cardioprotective and neuroprotective effects of moxibustion require long-term follow-up to fully evaluate. In this study, the follow-up period was limited to 12 weeks, which may be insufficient to capture the long-term benefits. Future research should include a longer prospective study with a follow-up period of up to 3 years to monitor major cardiovascular and cerebrovascular events, as well as patient self-management capabilities.

Adherence assessment for home-based moxibustion primarily relied on patient-reported measures. Although auxiliary methods, including random video verification, were used, objective monitoring of key parameters, such as moxibustion temperature, remained limited, potentially leading to overestimated adherence rates. Future studies should incorporate technological solutions, such as smart moxibustion devices with integrated temperature sensors and Bluetooth connectivity, or certified electronic diaries with time and geolocation verification. These innovations would enable automated objective data collection, substantially improving the quality of home-based TCM research.

The timing of patient enrollment also varied by season, which may have introduced seasonal variability in blood pressure outcomes. Future studies should consider recruiting participants during the same season to minimize this confounding effect. Additionally, the impact of moxibustion on blood pressure stability across different seasons in patients with hypertension remains an area for further investigation.

Despite these limitations, this study aims to provide high-quality clinical evidence for the efficacy of moxibustion and TCM-based lifestyle interventions in the treatment of hypertension and their protective effects on the heart and brain. The findings could have significant implications for the comprehensive prevention and management of chronic diseases.

## Appendix

Multimedia Appendix 1 Chinese Guidelines for the Prevention and Treatment of Hypertension [8] (2018 revision) standard: Increased systolic blood pressure (SBP) of ≥140 mmHg and/or diastolic blood pressure (DBP) of ≥90 mmHg should still be diagnosed as hypertension.

Multimedia Appendix 2 The measurement scale for the phlegm-dampness constitution in traditional Chinese medicine.

Multimedia Appendix 3 General information (cardiovascular risk stratification).

Multimedia Appendix 4 Moxibustion record form.

Multimedia Appendix 5 Moxibustion rating form.

## Abbreviations

ABPM: ambulatory blood pressure monitoring
DSMC: data safety monitoring committee
HF: high-frequency
HRV: heart rate variability
LF: low-frequency
SDNN: SD of normal-to-normal
TCM: traditional Chinese medicine

## Data Availability

Data sharing is not applicable to this paper as no data sets were generated or analyzed during this study.

## References

[1] GBD 2019 Risk Factors Collaborators (2020). Global burden of 87 risk factors in 204 countries and territories, 1990-2019: a systematic analysis for the Global Burden of Disease Study 2019. Lancet.

[2] GBD 2019 DiseasesInjuries Collaborators (2020). Global burden of 369 diseases and injuries in 204 countries and territories, 1990-2019: a systematic analysis for the Global Burden of Disease Study 2019. Lancet.

[3] NCD Risk Factor Collaboration (NCD-RisC) (2021). Worldwide trends in hypertension prevalence and progress in treatment and control from 1990 to 2019: a pooled analysis of 1201 population-representative studies with 104 million participants. Lancet.

[4] Wang Z, Chen Z, Zhang L, Wang X, Hao G, Zhang Z, Shao L, Tian Y, Dong Y, Zheng C, Wang J, Zhu M, Weintraub WS, Gao R, China Hypertension Survey Investigators (2018). Status of hypertension in China: results from the China hypertension survey, 2012-2015. Circulation.

[5] Choudhry NK, Kronish IM, Vongpatanasin W, Ferdinand KC, Pavlik VN, Egan BM, Schoenthaler A, Houston Miller N, Hyman DJ, American Heart Association Council on Hypertension; Council on CardiovascularStroke Nursing;Council on Clinical Cardiology (2022). Medication adherence and blood pressure control: a scientific statement from the American Heart Association. Hypertension.

[6] Kim CL, Do YS, Kim BJ, Lee K, Nah M, Kim U, Lee J, Hwang T (2021). Clinical impact of medication adherence on 10-year cardio-cerebrovascular mortality in newly diagnosed hypertensive patients. J Clin Hypertens (Greenwich).

[7] Alsaqabi YS, Rabbani U (2020). Medication adherence and its association with quality of life among hypertensive patients attending primary health care centers in Saudi Arabia. Cureus.

[8] Matos LC, Machado JP, Monteiro FJ, Greten HJ (2021). Understanding traditional chinese medicine therapeutics: an overview of the basics and clinical applications. Healthcare (Basel).

[9] Zhang H, Xia Z, Liu Y, Yu S, Shi H, Meng Y, Wu X (2024). Intervention of hypertension by acupuncture-related therapies: a network meta-analysis. Int J Older People Nurs.

[10] Li Z, Kong J, Yang F, Li W, Chen S, Wang J (2023). Application of Moxibustion for adjusting constitution in "Preventive Treatment of Diseases" of TCM. World Chinese Medicine.

[11] Zhou X, Xue Q, You J, Li S, Li L, Zhu W, Fu Y, Sun X (2023). Efficacy and safety of community-based moxibustion for primary hypertension: a randomized controlled trial with patient preference arms. J Clin Hypertens (Greenwich).

[12] Jiang X, Lu T, Dong Y, Shi J, Duan M, Zhang X (2022). Effectiveness and safety of moxibustion for vascular dementia: a systematic review and meta-analysis. Medicine (Baltimore).

[13] Cao S, Wang Z, Liu H, Ma X, Li L, Lu Y, Cheng L, Wu H (2023). Mechanism of moxibustion at Zusanli (ST36) in the treatment of hypertension: a review. World Chinese Medicine.

[14] Li P, Tjen-A-Looi SC, Cheng L, Liu D, Painovich J, Vinjamury S, Longhurst JC (2015). Long-lasting reduction of blood pressure by electroacupuncture in patients with hypertension: randomized controlled trial. Med Acupunct.

[15] Cheng L, Li P, Patel Y, Gong Y, Guo ZL, Wu H, Malik S, Tjen-A-Looi SC (2018). Moxibustion modulates sympathoexcitatory cardiovascular reflex responses through paraventricular nucleus. Front Neurosci.

[16] Li P, Longhurst JC (2010). Neural mechanism of electroacupuncture's hypotensive effects. Auton Neurosci.

[17] Li P, Tjen-A-Looi SC, Guo Z, Longhurst JC (2010). An arcuate-ventrolateral periaqueductal gray reciprocal circuit participates in electroacupuncture cardiovascular inhibition. Auton Neurosci.

[18] Cheng L, Li P, Tjen-A-Looi SC, Longhurst JC (2015). What do we understand from clinical and mechanistic studies on acupuncture treatment for hypertension?. Chin Med.

[19] Chan AW, Tetzlaff JM, Gøtzsche PC, Altman DG, Mann H, Berlin JA, Dickersin K, Hróbjartsson A, Schulz KF, Parulekar WR, Krleza-Jeric K, Laupacis A, Moher D (2013). SPIRIT 2013 explanation and elaboration: guidance for protocols of clinical trials. BMJ.

[20] Chinese Hypertension League, Chinese Society of Cardiology, Chinese Medical Doctor Association Hypertension Committee, Hypertension Branch of China International Exchange and Promotive Association for Medical and Health Care, Hypertension Branch of Chinese Geriatric Medical Association (2019). 2018 Chinese Guidelines for the Management of Hypertension. Chinese Journal of Cardiovascular Medicine.

[21] No author listed (2009). Classification and Determination of Constitution in TCM (ZYYXH/T157-2009). World Journal of Integrated Traditional and Western Medicine.

[22] Yan Y, Wang T (2020). Medical Statistics, 5th Edition.

[23] Wang Z, Yang Z, Chen A, Xie H (2022). Research progress on the correlation between TCM syndrome types and objective indexes of essential hypertension. Global Traditional Chinese Medicine.

[24] Xie H (2021). Correlation analysis of sputum evidence of hypertension, cardiovascular disease risk and cardiovascular prognosis index in patients with hypertension. Guangzhou University of Chinese Medicine.

[25] Minh HV, Tien HA, Sinh CT, Thang DC, Chen C, Tay JC, Siddique S, Wang T, Sogunuru GP, Chia Y, Kario K (2021). Assessment of preferred methods to measure insulin resistance in Asian patients with hypertension. J Clin Hypertens (Greenwich).

[26] Chen J, Chen X, Liao R, Sun X, Zhao X, Chen J (2024). Associations between traditional chinese medicine constitution types and prehypertension: single rate meta-analysis. World Chinese Medicine.

[27] Liu C, Liu Q, Zhang D, Liu W, Yan X, Zhang X, Oyanagi H, Pan Z, Hu F, Wei S (2018). Insight into the biological effects of acupuncture points by X-ray absorption fine structure. Anal Bioanal Chem.

[28] Lin JG, Kotha P, Chen YH (2022). Understandings of acupuncture application and mechanisms. Am J Transl Res.

[29] Liu S, Wang Z, Su Y, Qi L, Yang W, Fu M, Jing X, Wang Y, Ma Q (2021). A neuroanatomical basis for electroacupuncture to drive the vagal-adrenal axis. Nature.

[30] Yan X, Zhang X, Liu C, Dang R, Huang Y, He W, Ding G (2009). Do acupuncture points exist?. Phys Med Biol.

[31] Wu S, Chen W, Hsieh C, Lin Y (2014). Abundant expression and functional participation of TRPV1 at Zusanli acupoint (ST36) in mice: mechanosensitive TRPV1 as an "acupuncture-responding channel". BMC Complement Altern Med.

[32] Wang M, Cai H, Wu H, Yu Z, Wang X, Chen Z, Jin C, Zhang J (2016). Moxibustion in conduction response pathway between Zusanli and spinal cord. World Chinese Medicine.

[33] Wang J, Kario K, Chen C, Park J, Hoshide S, Huo Y, Lee H, Li Y, Mogi M, Munakata M, Park S, Zhu D (2018). Management of morning hypertension: a consensus statement of an Asian expert panel. J Clin Hypertens (Greenwich).

[34] Kuwajima I, Mitani K, Miyao M, Suzuki Y, Kuramoto K, Ozawa T (1995). Cardiac implications of the morning surge in blood pressure in elderly hypertensive patients: relation to arising time. Am J Hypertens.

[35] Li Y, Thijs L, Hansen TW, Kikuya M, Boggia J, Richart T, Metoki H, Ohkubo T, Torp-Pedersen C, Kuznetsova T, Stolarz-Skrzypek K, Tikhonoff V, Malyutina S, Casiglia E, Nikitin Y, Sandoya E, Kawecka-Jaszcz K, Ibsen H, Imai Y, Wang J, Staessen JA (2010). Prognostic value of the morning blood pressure surge in 5645 subjects from 8 populations. Hypertension.

[36] Julario R, Mulia EP, Rachmi DA, A'yun MQ, Septianda I, Dewi IP, Juwita RR, Dharmadjati BB (2021). Evaluation of heart rate variability using 24-hour Holter electrocardiography in hypertensive patients. J Arrhythm.

[37] Kario K (2005). Time for focus on morning hypertension: pitfall of current antihypertensive medication. Am J Hypertens.

[38] Kario K, Saito I, Kushiro T, Teramukai S, Tomono Y, Okuda Y, Shimada K (2016). Morning home blood pressure is a strong predictor of coronary artery disease: the HONEST study. J Am Coll Cardiol.

[39] Saylik F, Sarıkaya R (2021). Can systemic immune-inflammation index detect the presence of exxaggerated morning blood pressure surge in newly diagnosed treatment-naive hypertensive patients?. Clin Exp Hypertens.

[40] Chen M, Lin L, Chen R, Yang Y, Su J (2020). Association of early morning hypertension with target organ damage and 10-year risk of atherosclerotic cardiovascular disease. Chinese Journal of Hypertension 30.

[41] Stolarz-Skrzypek K, Thijs L, Li Y, Hansen TW, Boggia J, Kuznetsova T, Kikuya M, Maestre G, Mena L, Kawecka-Jaszcz K, Staessen JA (2011). Short-term blood pressure variability in relation to outcome in the International Database of Ambulatory blood pressure in relation to Cardiovascular Outcome (IDACO). Acta Cardiol.

[42] Castagna F, McDonnell BJ, Mondellini GM, Gaudig A, Pinsino A, McEniery C, Stöhr EJ, Takeda K, Naka Y, Uriel N, Yuzefpolskaya M, Cockcroft J, Parati G, Colombo PC (2022). Twenty-four-hour blood pressure and heart rate variability are reduced in patients on left ventricular assist device support. J Heart Lung Transplant.

[43] Hansen TW, Li Y, Boggia J, Thijs L, Richart T, Staessen JA (2011). Predictive role of the nighttime blood pressure. Hypertension.

[44] Hisamatsu T, Miura K, Ohkubo T, Arima H, Fujiyoshi A, Satoh A, Kadota A, Zaid M, Takashima N, Ohno S, Horie M, Ueshima H, SESSA Research Group (2018). Home blood pressure variability and subclinical atherosclerosis in multiple vascular beds: a population-based study. J Hypertens.

[45] Goldenberg I, Goldkorn R, Shlomo N, Einhorn M, Levitan J, Kuperstein R, Klempfner R, Johnson B (2019). Heart rate variability for risk assessment of myocardial ischemia in patients without known coronary artery disease: the HRV-DETECT (Heart Rate Variability for the Detection of Myocardial Ischemia) study. J Am Heart Assoc.

[46] Zhang X, Xu D, Shang Y (2024). The application value of 24-hour Holter monitoring in the diagnosis of asymptomatic myocardial ischemia. Journal of Practical Electrocardiology.

[47] Hill MA, Yang Y, Zhang L, Sun Z, Jia G, Parrish AR, Sowers JR (2021). Insulin resistance, cardiovascular stiffening and cardiovascular disease. Metabolism.

[48] Hou X, Lv Y, Li Y, Wu Q, Lv Q, Yang Y, Li L, Ye X, Yang C, Wang M, Cao L, Wang S (2024). Association between different insulin resistance surrogates and all-cause mortality in patients with coronary heart disease and hypertension: NHANES longitudinal cohort study. Cardiovasc Diabetol.

[49] Bello-Chavolla OY, Antonio-Villa NE, Vargas-Vázquez A, Martagón AJ, Mehta R, Arellano-Campos O, Gómez-Velasco DV, Almeda-Valdés P, Cruz-Bautista I, Melgarejo-Hernandez MA, Muñoz-Hernandez L, Guillén LE, Garduño-García JJ, Alvirde U, Ono-Yoshikawa Y, Choza-Romero R, Sauque-Reyna L, Garay-Sevilla ME, Malacara-Hernandez JM, Tusié-Luna MT, Gutierrez-Robledo LM, Gómez-Pérez FJ, Rojas R, Aguilar-Salinas CA (2019). Prediction of incident hypertension and arterial stiffness using the non-insulin-based metabolic score for insulin resistance (METS-IR) index. J Clin Hypertens (Greenwich).

