# Supplementary material for: Cardio-Cerebral Protective Effect of Moxibustion on Phlegm-Dampness Type Hypertension: Protocol for a Randomized Controlled Trial
Source: JMIR Res Protoc. 2025 Dec 29;14:e79158. doi: 10.2196/79158 (PMC12796880; doi:10.2196/79158)
Supplement: Multimedia Appendix 1 [file resprot_v14i1e79158_app1.docx]

Appendix 1

Refer to the"Chinese Guidelines for the Prevention and Treatment of Hypertension"^[8]^(2018 revision) standard:Increased systolic blood pressure(SBP)of≥140 mmHg and/or diastolic blood pressure(DBP)of≥90 mmHg,it should still be diagnosed as hypertension.

| Grade | SBP（mmHg) | DBP（mmHg) |
| --- | --- | --- |
| Grades 1 | 140-159 | 90-99 |
| Grades 2 | 160-179 | 100-109 |
| Grades 3 | ≥180 | ≥110 |
