# Supplementary material for: Cardio-Cerebral Protective Effect of Moxibustion on Phlegm-Dampness Type Hypertension: Protocol for a Randomized Controlled Trial
Source: JMIR Res Protoc. 2025 Dec 29;14:e79158. doi: 10.2196/79158 (PMC12796880; doi:10.2196/79158)
Supplement: Multimedia Appendix 2 [file resprot_v14i1e79158_app2.docx]

Appendix 2

The measuring scale for phlegm-dampness constitution of traditional Chinese medicine

| Experience/condition in the Past Year | No | Slightly | Sometimes | Often | All the time |
| --- | --- | --- | --- | --- | --- |
| Does your body feel heavy or lethargic? | 1 | 2 | 3 | 4 | 5 |
| Do you feel chest or stomach stuffiness? | 1 | 2 | 3 | 4 | 5 |
| Do you have oily skin on your forehead? | 1 | 2 | 3 | 4 | 5 |
| Do you upper eyelids swell larger than others (slight swelling at the upper eyelid)? | 1 | 2 | 3 | 4 | 5 |
| Does your mouth feel sticky? | 1 | 2 | 3 | 4 | 5 |
| Is your stomach/belly flabby? | 1 | 2 | 3 | 4 | 5 |
| Do you always have lots of  phlegm, especially in your throat? | 1 | 2 | 3 | 4 | 5 |
| Does your tongue have a thick coating? | 1 | 2 | 3 | 4 | 5 |

The original score is equal to the sum of the scores for each item, and the transformed score is calculated as follows: [(original score-number of items)/(number of items×4)]×100.When the transformed score≥40, it is phlegm-dampness constitution; scored 30 to 39,it tend to be phlegm-dampness constitution;＜30,it was not phlegm-dampness constitution.
