# Supplementary material for: Cardio-Cerebral Protective Effect of Moxibustion on Phlegm-Dampness Type Hypertension: Protocol for a Randomized Controlled Trial
Source: JMIR Res Protoc. 2025 Dec 29;14:e79158. doi: 10.2196/79158 (PMC12796880; doi:10.2196/79158)
Supplement: Multimedia Appendix 3 [file resprot_v14i1e79158_app3.docx]

Appendix 3:

General Information(Cardiovascular Risk stratification)

Name: Gender: Age:

1.History of hypertension?

2.Family history ofpremature cardiovascular disease? 3.Do you have hyperlipidemia?

4.Do you have diabetes?

5.Do you have Abdominal obesity? 6.Smoking status

( 1)No; (2)Yes,years of smoking?cigarettes per day?

Passive smoking(exposure to smoking for more than 15 minutes per day) 7.Alcohol consumption:(male>25g/day;female>15g/day)

( 1)No; (2)drinking alcohol; (3)former drinkers who have stopped drinking. 8.Dietary status

( 1)Reasonable diet,regular three meals; (2)High sodium and low potassium diet; (3)High oil and fat diet. 9.Sleep duration:average hours

( 1)<5 hours;(2)5-7 hours;And(3)more than 7 hours

10.Exercise:(4-7 times a week,each lasting 30-60 minutes)

( 1)Yes; (2)No

11.Stress Situations:

( 1)no pressure; (2)a little bit of pressure; (3)have definite pressure; (4)extreme stress; 12.Education level:

( 1)illiteracy; (2)primary school; (3)junior highschool; (4)highschool/technical secondary school;

(5)junior college or above 13.Comorbidities:

( 1)cerebrovascular disease; (2)cardiovascular diseases; (3)kidney disease; (4)peripheral vascular disease; (5)retinopathy; (6)fatty liver;

14.Target organ damage:

( 1)Left ventricular hypertrophy 1.No; 2.Yes;

(2)Carotid plaque: 1.No; 2.Not examined; 3.Yes.Specific circumstances:

(3)glomerular filtration

rate: 1.Normal; 2.abnormal(serum creatinine ;eGFR: ) Microalbuminuria: albumin/creatinine ratio:

Stratification of cardiovascular risk levels for hypertension:

（ 1 ）Low risk; (2)moderate risk; (3)high risk; (4)very high risk
