# Supplementary material for: Cardio-Cerebral Protective Effect of Moxibustion on Phlegm-Dampness Type Hypertension: Protocol for a Randomized Controlled Trial
Source: JMIR Res Protoc. 2025 Dec 29;14:e79158. doi: 10.2196/79158 (PMC12796880; doi:10.2196/79158)
Supplement: Multimedia Appendix 4 [file resprot_v14i1e79158_app4.docx]

Appendix 4:

Moxibustion record form

Name: Sex: Age:

| Date | | Moxibustion start and stop time | Adverse reactio |
| --- | --- | --- | --- |
| Week One |  |  |  |
|  |  |  |  |
|  |  |  |  |
| Week Two |  |  |  |
|  |  |  |  |
|  |  |  |  |
| Week Three |  |  |  |
|  |  |  |  |
|  |  |  |  |
| Week Four |  |  |  |
|  |  |  |  |
|  |  |  |  |
| Week Five |  |  |  |
|  |  |  |  |
|  |  |  |  |
| Week Six |  |  |  |
|  |  |  |  |
|  |  |  |  |
| Week Seven |  |  |  |
|  |  |  |  |
|  |  |  |  |
| Week Eight |  |  |  |
|  |  |  |  |
|  |  |  |  |
| Week Nine |  |  |  |
|  |  |  |  |
|  |  |  |  |
| Week Ten |  |  |  |
|  |  |  |  |
|  |  |  |  |
| Week Eleven |  |  |  |
|  |  |  |  |
|  |  |  |  |
| Week Twelve |  |  |  |
|  |  |  |  |
|  |  |  |  |
