# Supplementary material for: Cardio-Cerebral Protective Effect of Moxibustion on Phlegm-Dampness Type Hypertension: Protocol for a Randomized Controlled Trial
Source: JMIR Res Protoc. 2025 Dec 29;14:e79158. doi: 10.2196/79158 (PMC12796880; doi:10.2196/79158)
Supplement: Multimedia Appendix 5 [file resprot_v14i1e79158_app5.docx]

Appendix 5:

Moxibustion rating form

Name: Gender: Age:

|  | Moxibustion frequency | Moxibustion duration | Moxibustion localization | Moxibustion temperature | Adverse reaction | Total points |
| --- | --- | --- | --- | --- | --- | --- |
| Week One |  |  |  |  |  |  |
| Week Two |  |  |  |  |  |  |
| Week Three |  |  |  |  |  |  |
| Week Four |  |  |  |  |  |  |
| Week Five |  |  |  |  |  |  |
| Week Six |  |  |  |  |  |  |
| Week Seven |  |  |  |  |  |  |
| Week Eight |  |  |  |  |  |  |
| Week Nine |  |  |  |  |  |  |
| Week Ten |  |  |  |  |  |  |
| Week Eleven |  |  |  |  |  |  |
| Week Twelve |  |  |  |  |  |  |
| Total points |  | | | | | |

Moxibustion score will be performed weekly according to the patients' moxibustion records, moxibustion photos, videos and outpatient follow-up, and the total score will be calculated after 12 weeks oftreatment. The scoring details are as follows:

1. Moxibustion frequency: complete 3 times a week, 1 time every other day, 1 point for each completion, full score of 3 points;

2. Moxibustion duration: 45-60 minutes is appropriate, time is too short or too long are deducted 1 point, full score of 3 points;

3. Moxibustion site: moxibustion site accurate point selection score 1 points, full score 3 points;

4. Moxibustion temperature: moxibustion temperature control at 43 ~ 45 degrees Celsius is appropriate, the temperature is too low or too high, deduct 1 point, full score of 3 points;

5. Adverse reactions: If there are burns, blisters, dizzy moxibustion, ulceration, infection, local abscess and other conditions, or dizziness;

fatigue, allergy, numbness and other discomfort after moxibustion, deduct 1 point, and record in detail. If there is no adverse reaction, 1 point is scored out of 3 points;

6. The 100-point system is adopted, and the cumulative score of /1.8 in 12 weeks is the final score, with a full score of 100.
